# Lipid Metabolism: Immune Regulation and Therapeutic Prospectives in Systemic Lupus Erythematosus

**DOI:** 10.3389/fimmu.2022.860586

**Published:** 2022-03-18

**Authors:** Wei Sun, Pengchong Li, Jianping Cai, Jie Ma, Xuan Zhang, Yong Song, Yudong Liu

**Affiliations:** ^1^ Department of Rheumatology, Beijing Hospital, National Center of Gerontology, Institute of Geriatric Medicine, Chinese Academy of Medical Sciences, Beijing, China; ^2^ Department of Respiratory and Critical Care Medicine, Jinling Hospital, Medical School of Southeast University, Nanjing, China; ^3^ Department of Rheumatology and Clinical Immunology, The Ministry of Education Key Laboratory, Peking Union Medical College Hospital, Beijing, China; ^4^ Department of Gastroenterology, Beijing Friendship Hospital, National Clinical Research Center for Digestive Diseases, Beijing Digestive Disease center, Beijing Key Laboratory for Precancerous Lesion of Digestive Diseases, Capital Medical University, Beijing, China; ^5^ The Key Laboratory of Geriatrics, Beijing Institute of Geriatrics, Beijing Hospital, National Center of Gerontology, National Health Commission, Institute of Geriatric Medicine, Chinese Academy of Medical Sciences, Beijing, China; ^6^ Center of Biotherapy, Beijing Hospital, National Center of Gerontolog, Beijing, China; ^7^ Institute of Geriatric Medicine, Chinese Academy of Medical Sciences, Beijing, China; ^8^ Department of Respiratory and Critical Care Medicine, Affiliated Jinling Hospital, Medical School of Nanjing Medical University, Nanjing, China

**Keywords:** systemic lupus erythematosus (SLE), dyslipidemia (DLP), lipid metabolism, immunocyte, autoimmunity

## Abstract

Systemic lupus erythematosus (SLE) is a heterogeneous disease characterized by the production of abnormal autoantibodies and immune complexes that can affect the organ and organ systems, particularly the kidneys and the cardiovascular system. Emerging evidence suggests that dysregulated lipid metabolism, especially in key effector cells, such as T cells, B cells, and innate immune cells, exerts complex effects on the pathogenesis and progression of SLE. Beyond their important roles as membrane components and energy storage, different lipids can also modulate different cellular processes, such as proliferation, differentiation, and survival. In this review, we summarize altered lipid metabolism and the associated mechanisms involved in the pathogenesis and progression of SLE. Furthermore, we discuss the recent progress in the role of lipid metabolism as a potential therapeutic target in SLE.

## Introduction

Systemic lupus erythematosus (SLE) is a complicated autoimmune disease characterized by various immune defects and chronic inflammation affecting multiple tissues and organs, and with a remarkably high morbidity and mortality rate (1). Immunological abnormalities, including the production of abnormal autoantibodies directly against various components, deposition of complement-fixing immune complexes, and dysregulation of immune cells, are the major hallmarks of SLE pathogenesis. Different types of immunocytes such as dendritic cells (DCs), macrophages, and neutrophils have been implicated as key players in SLE (2). However, the complex mechanisms underlying this systemic autoimmune disease remains to be elucidated (3, 4).

Although the exact mechanisms of autoimmune responses in SLE are not well understood, rapid development of technologies offers opportunities to examine various aspects of the diseases, providing novel insights into their pathogenesis. Lipidomics, as an independent branch of metabolomics, can be used to identify temporal and spatial changes in the lipid profile, thus, unravelling the complicated etiologies at the molecular level (5–7). Multiple changes in the lipid profiles have been documented in various metabolomic studies. Lipids are composed of various types of water-insoluble molecules, including cholesterol, fatty acids, oxysterols, triglycerides, and sphingolipids, which are essential components of biological membranes and organelles (8, 9). Regulation of lipid metabolism, including lipid uptake, lipogenesis, lipolysis, and its mediators, is important for cellular homeostasis. In addition to serving as building blocks of membranes, lipids also function as sources of energy, as well as signaling molecules or secondary messengers and participate in the modulation of diverse pathophysiological processes, including cell proliferation, differentiation, migration, activation, and survival (10). Thus, imbalances in lipid homeostasis can result in toxicity and contribute to SLE (11).

Growing evidence suggests lipid-mediated activities in immunocytes and the critical role of lipid metabolic dysregulation in the pathogenesis and progression of SLE. However, the mechanisms through which lipid metabolism affects the immune system remain unclear. In this review, we summarize the growing advances in lipid metabolism in SLE that could facilitate the development of novel therapeutic targets.

## Lipid Metabolism

**Figure 1:**
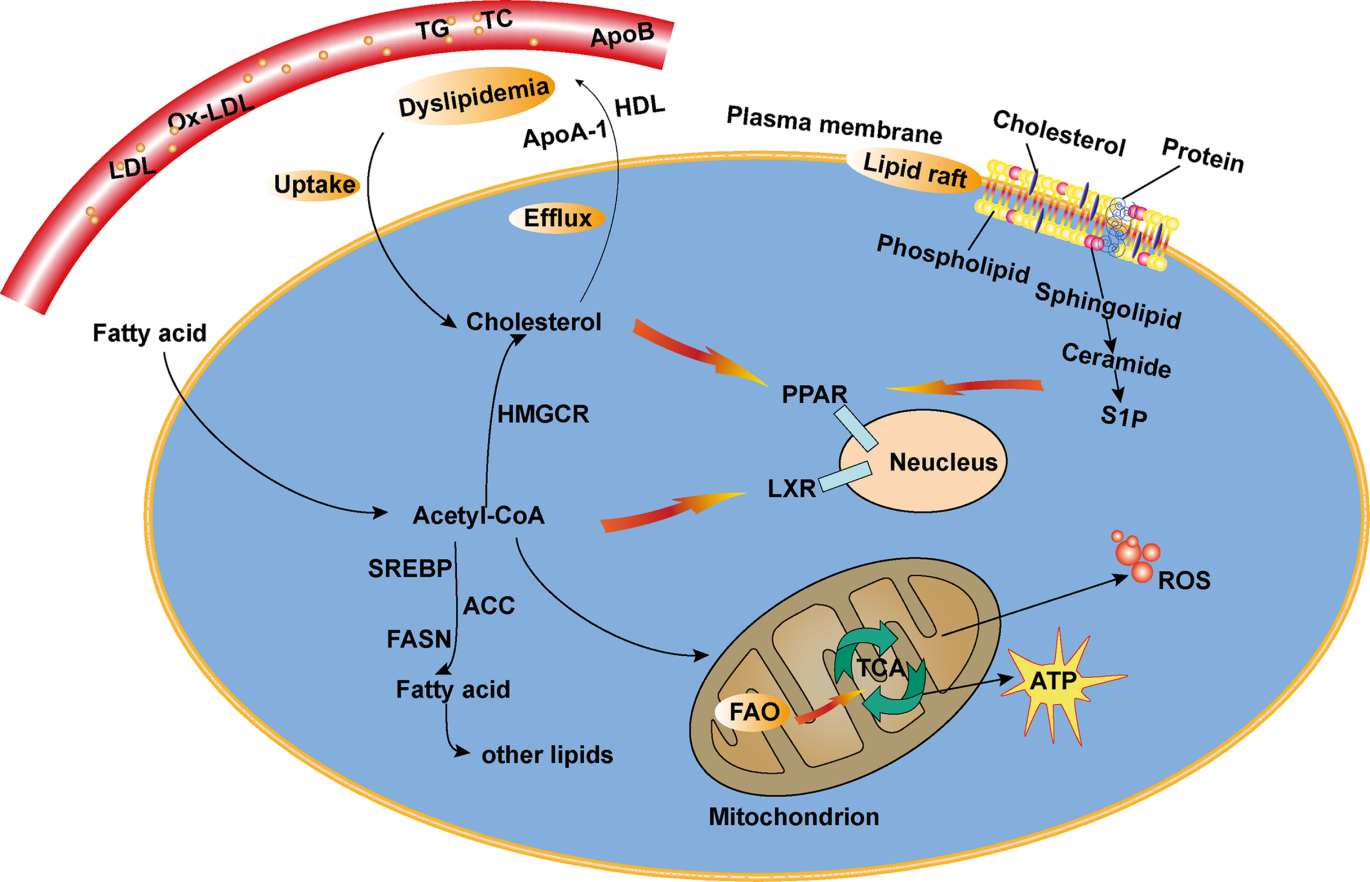
A simplified schematic representation of lipid metabolism. Lipid metabolic networks mainly involve catabolism (fatty acid oxidation (FAO)), anabolism (*de novo* lipogenesis) and storage as lipid droplets (LDs). Lipid raft are microdomains of the plasma membrane enriched in sphingolipids and cholesterol. The uptake of various lipids undergoes a series of processes and oxidation in the mitochondrion, which leads to the production of energy (substantial ATP) and ROS. Acetyl CoA, the end product of FAO pathway, can then either enter TCA cycle or synthesize multiple lipids, such as cholesterol and fatty acids. Lipids or derivatives of the lipid metabolism can activate transcription factors, including PPAR, LXR, then regulate activities of transporters to modulate downstream signals. Dyslipidemia, the disruption of lipid metabolism, is mainly characterized by elevated plasma levels of LDL, TG), TC), and reduced HDL.

Lipid metabolism, including anabolism and catabolism, is essential for almost every aspect of cellular funtioning (**Figure 1**). The fatty acid (FA) and cholesterol metabolic pathways are the most important pathways in lipid metabolism. A wide range of lipids, such as FAs, cholesterol, triglycerides, and steroids, participate in lipid synthesis critical for the cell (12). FAs are indispensable for lipid biosynthesis, the process of which involves multiple lipogenic enzymes as shown in **Table 1**, such as ATP citrate lyase, fatty acid synthase (FASN), acetyl-CoA carboxylase (ACC), and 3-hydroxy-3-methylglutaryl CoA reductase (HMGCR). Cholesterol, together with newly synthesized phospholipids and glycosphingolipids (GSLs), are critical components of the cell membrane microdomains called lipid rafts (45). These lipid rafts can coordinate interactions between key signaling molecules in order to regulate downstream transduction events, including modulation of antigen receptor signaling-mediated responses and the phosphoinositide 3-kinase (PI3K) pathway in SLE (46, 47). Liver X receptors α (LXRα) and β (LXRβ) are members of the nuclear receptor superfamily that are implicated in regulating lipid metabolism, including cholesterol, FAs, and phospholipids (48, 49). Furthermore, LXR maintains steady-state levels of various lipids by regulating the expression of sterol regulatory element-binding protein (SREBP), which is critical for lipogenesis (13, 14).

**Table 1:** Key genes involved in lipid metabolism of SLE.

Protein	Abbreviation	Functions in lipid metabolism	Participation in SLE	References
Sterol regulatory element-binding protein	SREBP	A lipogenetic enzyme	coordination of lipid synthesis and relevant receptors in cells	(13, 14)
Acetyl CoA		A lipid mediator	Alters lipid profiles and drives the TCA cycle for energy production	(15–17)
Peroxisome proliferator-activated receptor	PPAR	A transcription factor control genes involved in lipid metabolism	Affects immunocytes differentiation and functions, including production of cytokines and antibodies, modulates inflammatory signals	(18–23)
Acetyl-CoA carboxylase	ACC	A rate-limiting in *de novo* lipogenesis and connects energy metabolism	Associated with differentiation and responses of T cells	(17, 24–27)
Fatty acid synthase	FASN	A lipogenetic enzyme	Associated with death and differentiation of immunocytes as well as cytokines production, such as IFN-γ	(28, 29)
AMP-activated protein kinase	AMPK	A key signal in lipid metabolic pathways	Modulates inflammatory and immune responses	(30, 31)
mammalian Target of rapamycin	mTOR	A key signal in lipid metabolic pathways	Important for the proliferation, activation and differentiation of immunocytes, associated with disease activity as well	(32–36)
Liver X receptor	LXR	A nuclear receptor controls cellular lipid metabolism	Facilitates macrophage cholesterol efflux, affects acquired immune responses, its polymorphisms are related to SLE patients	(37–41)
ATP-binding cassette transporters A1 and G1	ABCA1/G1	Mediation of the cholesterol efflux	Associated with functions of dendritic cells	(42)
Glutathione peroxidase 4	GPX4	Associated with lipid peroxidation and ferroptosis	Downregulated in neutrophils and associated with disease activity	(43, 44)

In addition to lipid synthesis, lipid degradation is an important part of lipid metabolism, which is essential to sustain life. Lipolysis, the catabolic branch of the FA cycle, mainly refers to the process by which long-chain FAs are metabolized into acetyl-CoA (15). FAs or their derivatives can bind to and activate certain transcription factors. One of the most well-known transcription factors known to play a role in lipid metabolism is the peroxisome proliferator-activated receptor (PPAR) family, which consists mainly of PPARα, PPARγ, and PPARδ, also known as PPARβ (18). The accumulation of cellular lipids contributes to the activation of these transcription factors, including PPARγ and LXRs, and regulates the activities of transporters in order to modulate the efflux of free cholesterol and scavenger receptors. FAs generated by lipolysis are broken down through the fatty acid β-oxidation pathway in mitochondria, leading to the production of energy (ATP) and reactive oxygen species (ROS) (50). Fatty acid oxidation (FAO) is a mitochondrial aerobic process that converts FAs into multiple lipid mediators such as acetyl-CoA, which drives the tricarboxylic acid (TCA) cycle for energy production (16).

Lipid droplets (LDs) are ubiquitous organelles of lipid storage that serve as centers for lipid metabolism. The storage of neutral lipids in LDs provides energy and lipids for membrane lipid synthesis, which is critical for cell proliferation and remodeling (51). Moreover, LDs can prevent the accumulation of toxic lipids in the endoplasmic reticulum (52). The endoplasmic reticulum is an important organelle that participates in multiple physiological processes, including lipid synthesis (53). Lipid metabolism is an intricate process that generates multiple biological mediators. Many of these molecules are bioactive lipids involved in multiple signaling networks, participating in the development and progression of many inflammatory and autoimmune disorders, including SLE (54).

It is now known that lipidomics (also designated lipid profiling) can provided further insights into lipid metabolism that are beneficial to the advancement of medical research (55). In addition, lipoproteins, a group of biochemical assemblies that contain lipids and proteins, can serve as enzymes, transporters, and antigens capable of regulating multiple cellular activities. Distinct apolipoproteins regulate the metabolism of lipoproteins by participating in the transport and redistribution of lipids among cells and organs (56).

## Dyslipidemia in SLE

Dyslipidemia refers to the disruption of lipid metabolism and is mainly characterized by elevated plasma levels of low-density lipoprotein (LDL), triglyceride (TG), and total cholesterol (TC) levels, as well as reduced high-density lipoprotein (HDL) levels. Dyslipidemia is believed to be involved in disease pathogenesis, especially SLE (57). Cumulative evidence strongly supports the hypothesis that patients with SLE are prone to cardiovascular complications (58). The prevalence of ischemic heart disease in SLE patients ranges from 3.8 to 16%, which is close to 10-fold higher than that observed in the general population, and 50-fold higher in young women of reproductive age (59). The prevalence of dyslipidemia is significantly higher in a retrospective study of patients with SLE than that in otherwise healthy people (60). The prevalence of dyslipidemia ranges from 68-100% among adults with SLE (61). In the Systemic Lupus International Collaborating Clinic cohort study of newly diagnosed patients with SLE (n=198), the prevalence of hypercholesterolemia was 36% (60). A 6-year population-based cohort study demonstrated that children with SLE were more susceptible to heart failure and dyslipidemia than those without SLE (62). Additionally, a cross-sectional controlled study which evaluated 33 adolescent girls with juvenile SLE found nearly half patients had dyslipidemia, including decreased HDL-c concentrations; besides, juvenile patients showed decreased concentrations of apolipoprotein A-1 (ApoA-1) and ratio of LDL/apolipoprotein B (ApoB), all of which could increase atherosclerotic risk (63). Dyslipidemia had a higher prevalence among young female patients with SLE than among controls; this was characterized by decreased TC, LDL-c, HDL-c, ApoA, and ApoB. Univariate correlational analyses exhibited complicated correlations between serum levels of certain lipids and the SLE disease activity (64). A study recruited 71 female SLE patients and found that the plasma TG level is an independent predictor of carotid atherosclerosis in women with SLE (65). In addition, dyslipidemia could affect the prognosis of SLE patients not only through CVD-related disorders but also injuries to other organs, such as lupus nephritis (LN) (66). Aberrant lipoproteins are strongly associated with abnormal renal function in chronic kidney diseases (67). Dyslipidemia is frequent and more serious in LN patients than in those with a similar degree of other chronic kidney diseases (68). High frequency of lipid abnormalities, including increased levels of TG (58.5%), TC (55.4%), LDL-c (30.8%), and lower levels of HDL (21.5%), which were strongly related to their proteinuria were found in patients with LN (69). Dyslipidemia, such as elevated TC levels, not only reflects the activity but also the severity of renal injury (70, 71). Overall, dyslipidemia as a significant risk factor can result in renal failure and higher mortality in patients with LN (70). Notably, systemic corticosteroids when used at high doses or for a prolonged duration, lead to adverse events such as immunosuppression and dyslipidemia.

Low HDL levels are one of the most common dyslipidemia observed in patients with SLE (72). It is a consensus that HDL-c, which can be routinely detected in clinical practice, is considered synonymous with HDL particle levels. Lupus HDL promotes pro-inflammatory responses through activation of the nuclear factor kappa B (NF-κB) (73). Serum cholesterol efflux capacity is impaired in SLE, with a specific mechanism pattern in each disease, independent of serum HDL levels (74). Another study found a higher prevalence of dyslipidemia in patients with SLE (46.2%) than that in controls (19.2%); besides, the patient group had higher Cys levels and lower HDL-c levels compared with the control (75). ApoA-1 is the main protein fraction of HDL and can stabilize paraoxonase-1 (PON-1), which protects LDL from oxidation (76). The ratio of baseline ApoB : ApoA-1 has been shown to correlate negatively with a low disease activity, accordingly, positively with the SLE disease activity index (SLEDAI) over a 5-year follow-up period (77). Elevated levels of antibodies against HDL and ApoA1 have been reported in patients with cardiovascular disease (CVD), and are significantly higher in patients with SLE, the levels of which were negatively correlated with the activity of paraoxonase (PON) (78). Moreover, PON1 arylesterase activity and total HDL antioxidant capacity are significantly decreased in patients with SLE compared with that in controls. All HDL sub-fraction levels and HDL antioxidant abilitiy were positively correlated with PON1 arylesterase activity and negatively correlated with disease activity as well as the examined inflammatory markers of interest, such as hsCRP and IL-6 (72). In autoimmune mouse models, autoantibodies against ApoA-1 contribute to a reduction in HDL-c levels and a lower PON1 activity independent of hepatic HDL synthesis (79). Additionally, studies have found negative correlations between serum HDL-c levels and disease activity. Two dyslipidemia patterns are evident in pediatric active SLE, characterized by elevated TG and low HDL levels (80). Patients with active disease show lower levels of HDL, which can be elevated after the use of prednisone and hydroxychloroquine (81). A study aimed to identify potential biomarkers of disease activity by analyzing the proteome of HDL particles in patients with SLE, the authors found that the plasma gelsolin levels decreased significantly in patients and were significantly associated with HDL-c levels, especially when patients developed a clinical flare, suggesting an association between dyslipidemia and disease activity (82).

A cohort study reported that, in pediatric SLE patients, higher LDL levels were associated with disease activity (83). Ricardo et al. obtained circulating LDL particles from SLE patients and found that LDL from a flare was more atherogenic and induced greater endothelial cell migration than LDL from inactive patients (84). Lipids are highly susceptible to oxidation, and products of lipid peroxidation are potential biomarkers suggestive of oxidative stress condition in SLE (85). The oxidative modification of LDL is an earlier event in atherosclerosis. Oxidized LDL (ox-LDL) is a common lipid peroxidation marker, represents various modifications of lipids by lipid peroxidation. Patients with SLE (especially those with CVD) show more oxidized epitopes on LDL than controls, and anticardiolipin antibodies in these patients recognize epitopes produced during lipid peroxidation (86, 87). Moreover, cross-reactivity was found between antiphospholipid antibodies, which are closely related to thrombosis, and antibodies to ox-LDL in SLE (88). More specifically, an increase in plasma levels of L5 (a sub-fraction of electronegative LDL), but not total LDL concentrations, may contribute to early vascular aging and premature atherosclerosis in SLE patients (89). SLE patients had higher levels of circulating ox-LDL, and patients with dyslipidemia were given a higher cumulative prednisolone dose than controls (90). Taken together, higher LDL and ox-LDL levels are positively correlated with a higher risk of cardiovascular complications in patients with SLE. An amount of HDL is dysfunctional and can not inhibit the oxidation of LDL in SLE patients.

Of note, as a conventional treatment in SLE, corticosteroids, particularly when used at high doses or prolonged duration, leads to adverse events such as immunosuppression and dyslipidemia. Corticosteroid-treated female patients with SLE have manifestations of higher levels of plasma ApoB as well as hyperlipidemia including VLDL, TG and LDL (91). While, among juvenile SLE, the cumulative corticosteroid dose was associated with a decrease in HDL-c (92). Interestingly, glucocorticoids regulate both lipid synthesis and degradation under different circumstances. The metabolic functions of glucocorticoid are mediated by the glucocorticoid receptor, a nuclear hormone receptor expressed various kinds of cells (93). Many mediators of the glucocorticoid receptor have been identified to regulate the lipid metabolism. Moreover, mechanisms of dyslipidemia induced by steroids are complex, including hepatic insulin resistance, higher levels of lipogenesis enzymes such as acetyl-CoA carboxylase and free fatty acid synthetase, lower levels of lipoprotein lipase and LDL receptor (94, 95). Therefore, if hormones are needed in long-term or large amount for SLE treatment, monitoring lipid profiles in blood are recommended. Notably, as the cornerstone in the treatment of SLE, antimalarials- hydroxychloroquine was demonstrated in a longitudinal study to exert favorable effects on lipids in SLE since the 3-month of hydroxychloroquine induced a significant reduction of TC and LDL, which determined an obvious decrease in the frequency of dyslipidemia (96). Similarly, a meta-analysis quantitively measured the beneficial effect of hydroxychloroquine on serum LDL levels in patients with SLE, and pooled data showed that hydroxychloroquine could lead to a reduction of mean LDL levels by 24.397 mg/dL (P = 0.002) (97). The effects of antimalarials on lipid profiles partially may because of an overall reduction in hepatic lipid synthesis (98). Besides, there is evidence that the lower levels of LDL induced by chloroquine in SLE are associated with the upregulation of LDL receptors (99). Hence, the benefits of hydroxychloroquine treatment in SLE not only because of its direct effects on disease activity but also its indirect effects on lipids to protect against dyslipidemia, especially those with higher cardiovascular risk or treated with glucocorticoids (100). While, dyslipidemia in SLE could also be induced by other immunosuppressant agents. Calcineurin inhibitors (e.g., tacrolimus and cyclosporine) widely used as immunosuppressants in autoimmune diseases especially in lupus nephritis, have side effects of hyperlipidemia as well (101). Specifically, cyclosporine could lead to hyperlipidemia including increased levels of TG, TC, LDL and Apo-B, partially through inhibiting certain hydroxylases and lipoprotein lipase (102). Besides, cyclosporine downregulates the LDL receptors and disturbs their binding of LDL-c, resulting in a decrease in cholesterol and LDL clearance respectively (95). What’s more, a novel calcineurin inhibitor, voclosporin, was approved for the treatment of adult patients with active lupus nephritis by the US Food and Drug Administration last year, exerts a more beneficial effect on lipid profiles such as lower levels of cholesterol and LDL than tacrolimus (103, 104). Meanwhile, another widely used immunosuppressant, cyclophosphamide can also reduce serum levels of LDL and VLDL in lupus nephritis patients (105). Overall, further explorations are needed to measure clinical benefits and lipid metabolic effects of these common drugs used in clinical practice.

## Lipid Metabolism in Immunocytes in SLE

**Figure 2:**
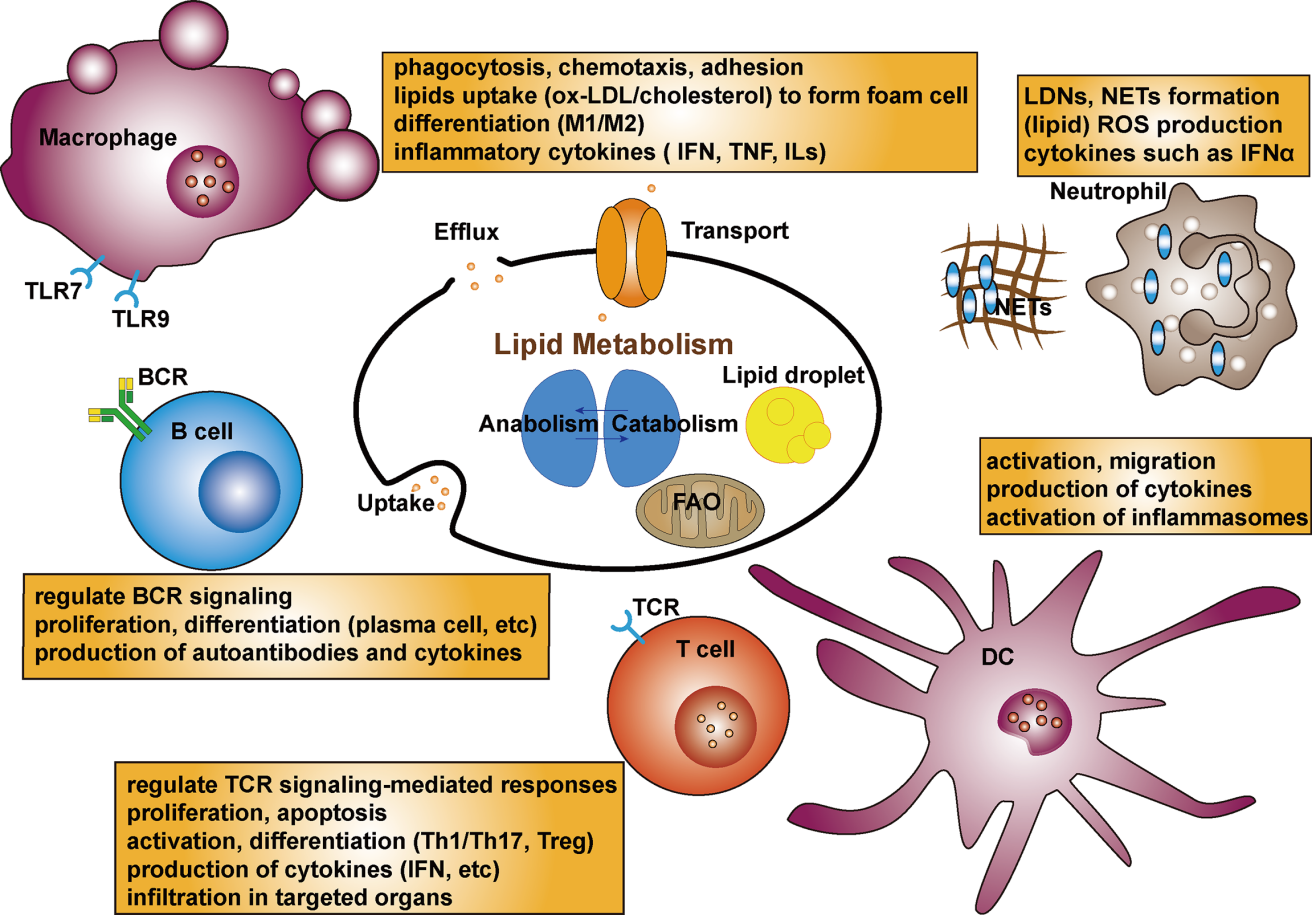
Schematic diagram of the lipid metabolism in immunocytes in SLE. Lipid rafts can regulate TCR signaling-mediated responses. Lipid metabolism as an important facilitator of T cell differentiation, can activate CD4+T cells then differentiate into Th or Treg cells. Lipid biosynthesis including cholesterol and fatty acid is critical for proliferation, apoptosis and production of cytokines in T cells. Dyslipidemia can promote the autoantibodies in B cells. Lipid rafts also regulate BCR signaling. Lipids composition could affect B cell proliferation, differentiation and production of autoantibodies as well as interleukins. Fatty acids can modulate the process of macrophage differentiation as well as adhesion on endothelial cells and their chemotaxis. The uptake of ox-LDL by macrophages uptake by the macrophage is the initial step to foam cell formation. Targeting receptors associated lipid metabolism can affect differentiation of monocytes and production of proinflammatory cytokines, which are related to TLR7 and TLR9 expression. Lipid profiles promote activation and migration of DCs. LDLs promote the production of IL-6 in DCs. Besides, cholesterol-enriched DCs show augmented cytokine secretion and activation of inflammasome. A subset of lupus proinflammatory neutrophils (LDGs) increase and promote risk of atherosclerosis. Oxidation of cholesterol and phospholipids can lead to lipid peroxidation, and higher level of ROS production with enhanced catalase activity of neutrophils are seen in SLE. NETs stimulate production of IFNs and proinflammatory cytokines. NETs possess active oxidant-generating enzymes, and modify HDL.

Current evidence suggests that lipids and lipid metabolites play an important role in the immune system by directly acting on immune cells through indirect modulation of antigen presentation and cytokine production (106). In view of the emerging role of lipid metabolism in autoimmune disorders, an increasing number of studies are exploring lipids in immunocytes in SLE (**Figure 2**). Aberrant immunity with inflammatory mediators and dysfunctional immune cells, especially the loss of T- and B-cell tolerance to self-antigens, are the major characteristics of SLE pathogenesis (107, 108). It has been reported that compared with healthy controls, lipidomics analysis of peripheral blood mononuclear cells from SLE patients revealed altered lipid metabolism, including significantly increased lysophospholipids, decreased plasmalogens, and altered phosphatidylserines (109).

### Lipid Metabolism in Lymphocytes in SLE

T cells, especially CD4+T cells, are important in driving B cell production of abnormal autoantibodies and are central to SLE pathogenesis (110). Abnormal lipid metabolism can impair T-cell function in SLE. Lipid rafts can regulate T-cell antigen receptor (TCR) signaling-mediated responses in T cells. Lipid raft analysis demonstrated that cholesterol efflux mediated by HDL and ApoA-1 could link immunity and cardiovascular protection. ApoA-1 and HDL decreased cholesterol and major histocompatibility class II protein content, indicating that cholesterol efflux from antigen-presenting cells to HDL and ApoA-1 could block antigen presentation and T-cell activation by decreasing lipid raft assembly (111). A study indicated that increased FcR and activated Syk kinase in SLE T cells have more ganglioside-containing lipid rafts; meanwhile, cross-linking of lipid rafts could promote earlier and stronger calcium responses (112). Therefore, changes in lipid rafts can strengthen and promote T-cell activities in SLE. Furthermore, aggregation of lipid rafts and rewiring of the CD3 complex grants T cells a lower activation threshold and distorts downstream signalings, consequently directing the abnormal expression of adhesion molecules in aggregated lipid rafts to relavant targeted organs (113). Treatment targeting lipid raft-associated signaling has been demonstrated to restore defects in T cell activation, and reduce the production of pro-inflammatory cytokines in lupus auto-reactive T cells, which provides a rationale for further research (114). Glycosphingolipids (GSLs), which are composed of a ceramide backbone, are enriched in lipid rafts. Increased GSL synthesis is a feature of T cells in SLE and is closely related to TCR activation. CD4+ T cells from patients with SLE show an aberrant profile of lipid raft-associated GSLs compared with that from volunteers, and elevated GSLs are suggested to be associated with overexpression of LXRβ, a nuclear receptor mentioned before affects acquired immune responses as well (115). In addition to its influence on CD4+ T cell signaling and function, the inhibition of GSL biosynthesis *in vitro* can decrease the production of anti-dsDNA antibody by activated B cells. Similarly, decreasing the levels of GSLs in CD4+ T cells led to a moderation of the T cell response toward activation; concomitantly, dramatic decreasing IL-2 production and T-cell proliferation have been observed as well (116). In lymphocytes, the synthesis of GSLs is mainly controlled by the transcription factor Friend leukemia integration 1 (Fli1), which can affect the function of immune cells and implicated in the pathogenesis of autoimmune disorders (117). A significant decrease in the number of CD4+T cells was observed in the kidneys of MRL/lpr Fli1(+/-) mice compared with that of Fli1(+/+) nephritic mice; reducing Fli1 in lupus-prone mice would protect against lupus nephritis partially by regulating CXCR3 expression on T cells and reducing T-cell infiltration of kidneys (118).

Lipid metabolism is increasingly recognized as an important facilitator of T cell differentiation. Activated CD4+T cells can differentiate into T helper (Th) cells or inducible T regulatory (Treg) cell subsets with distinct immunological functions. Lipid biosynthesis, including cholesterol and fatty acids, is critical for the proliferation and differentiation of T cells, especially a subset of Th17 cells (119). Th17 cells are featured by the expression of their signature cytokine IL-17A and the master transcription factor retinoid-related orphan receptor (120). Acetyl-CoA is an important metabolite involved in the synthesis, degradation, and remodeling of various lipids, thus altering lipid profiles. Acetyl-CoA carboxylase 1 (ACC1) associates key energy metabolism with lipogenesis and is a rate-limiting enzyme involved in *de novo* lipogenesis through the carboxylation of acetyl-CoA (17). Most *de novo* synthesized fatty acids are incorporated into phospholipids or localized to lipid rafts to be implicated in membrane-related activities, and some are stored in the form of lipid droplets to buffer excess lipids. T cells deficient in specific ACC1 with impaired lipid synthesis show reduced potential for differentiation of naive CD4+T cells into both Th1 and Th17 cells and higher proportions of Treg cells (24). Regulation of *de novo* fatty acid synthesis by targeting ACC1 in T cells could be therapeutic for autoimmune and inflammatory diseases (25, 26). Mice with a deletion of ACC1 specifically in T cells, failed to respond efficiently and were susceptible to infection, suggesting the critical role of ACC1-dependent fatty acid synthesis in T cells to fight exogenous infection (27). Voss et al. explored the effects of lipid metabolism on apoptosis sensitivity in T cells, showing that inhibition of FASN protected human CD4+T cells from restimulation-induced cell death (RICD), the process of which relied on sphingolipid synthesis and FAO (28). FASN, downstream of ACC, is a key lipid metabolic regulator for the generation of inflammatory Th17 cells, and inhibition of FASN contributes to the production of IFN-γ by Th17 cells (29). Studies in both human and murine models have indicated the essential role of Th17 cells in SLE pathogenesis; thus, targeting mediators implicated in the regulation of Th17 would be meaningful (121). The CD95 ligand (CD95L) is expressed by various immunocytes and can trigger apoptosis. Metalloprotease-cleaved CD95L (cl-CD95L) is implicated in homeostasis of the immune system (122). Th17 cells stimulated with cl-CD95L could produce sphingosine 1-phosphate (S1P), a potent lipid mediator distributed to HDL, through which Th17 cells transmigrate across the endothelial barrier; thus, exploring the CD95 relevant signaling pathway could be therapeutic for SLE patients (123, 124). Inhibiting FA synthesis in memory CD4+T cells of SLE patients decreased interferon-γ production and increased Foxp3 expression in T-bet+Foxp3+ cells (125).

Oxidative stress is augmented in patients with SLE, as indicated by increased products of lipid peroxidation such as malonaldehyde (MDA) and decreased antioxidants such as glutathione, which are implicated in the regulation of the immune system (126). AMP-activated protein kinase (AMPK), an important regulator in metabolism, is a rate-limiting enzyme in promoting FAO, while AMPK-dependent ACC phosphorylation can inhibit *de novo* lipogenesis (30). Treg cells activate AMPK and are dependent on lipid oxidation (31). In T cells, the dysfunction of mitochondrion promotes lipid hydroperoxides release, which spread oxidative stress to other organelles and peripheral blood (127). A case-control study enrolled 204 SLE patients and 256 healthy volunteers and measured their lipid peroxides and found that increased peroxides were associated with a Th1 and Th17 immune shift (128). Moreover, AKT/mTOR signaling is important in SLE and is activated in T cells of patients (32). It is known that in activated immune cells, mTOR promotes lipid synthesis and is critical for the differentiation of CD4+T and CD8+T cells (33). Recently, a cohort of juvenile-onset SLE patients found that patients with a higher ratio of ApoB : ApoA1 exhibited higher frequencies of CD8+T cells; besides, a CD8+T-cell transcriptomic profile enriched in genes related to atherosclerosis, including those related to interferon signaling (77). Invariant natural killer T (iNKT) cells, which exhibit defective autoimmunity can promote atherosclerosis through CD1d-mediated lipid antigen presentation, and link lipids with immune responses in SLE patients with CVD (129). The inhibitory receptor B and T lymphocyte attenuator (BTLA) can negatively modulate immune responses. The BTLA signaling pathway is altered in SLE T cells, and the impaired capacity of BTLA can be corrected by normalizing the lipid metabolism in lupus CD4+ T cells (130).

Emerging evidence suggests that B cells in autoimmunity are accompanied by significant shifts in metabolic processes, thus determining the mechanisms of this new area of active study.

In animal models of SLE, hyperlipidemia promoted the production of autoantibodies in B cells by inducing autoimmune CXCR3+ follicular helper T cells (131). However, studies involving the role of lipid metabolism in B cells in SLE patients is limited relative to T cells. Defective control of cellular signals in lymphocytes, such as B-cell receptor (BCR) signaling, could directly result in autoimmunity in SLE (132). Assessment of B cells from patients with SLE showed larger stained lipid rafts, and lower molecular weight isoform of CD45 in lipid rafts compared with healthy controls; the abnormal CD45 translocation is associated with the dynamics of Lyn (a negative regulator of BCR signaling) in lipid rafts and BCR–antigen contact regions (133). Similarly, purified lipid raft signaling domains in B cells collected from a series of SLE patients showed lower Lyn levels and abnormal translocation to lipid rafts, and these changes in Lyn were in relation to increased spontaneous proliferation, production of anti-dsDNA auto-antibodies and cytokines (134). Apart from the importance of LXRs in lipid metabolism, they can modulate functions of immunocytes as well (37). Pharmacological activation of LXRs ameliorates disease activity in lupus-prone mice (38). The authors found that a cholesterol-containing diet in ApoE/LXRβ-deficient mice would result in lupus-like disease, and defects of LXRβ in CD11c+cells led to B-cell expansion (39). In addition to T cells, over-activation of mTOR signaling in B cells also relates to plasmablast counts and SLE activity (34). Evidence suggests that the mTORC1 pathway is prominently activated in lupus Atypical memory B cells (AtMs), and blocking mTORC1 signaling significantly reduces the terminal differentiation of AtMs (35). Mechanisms of mTOR signaling are complicated by B cell formation, activation, and differentiation in SLE. Lupus-prone mice treated with mTOR inhibitors showed a reduction in B cells differentiation into germinal centers and plasma cells, and lower disease activity (36). Recently, a study suggested a molecular link between the dysregulation of lipid metabolism and the pathogenesis of lupus (135). The authors collected B cells from patients with lupus and found that inositol-requiring enzyme 1α was positively correlated with the amount of B cell lipid deposition, demonstrating that this enzyme could control plasma cell differentiation by modulating the coenzymes associated with fatty acid synthesis. Fatty acid composition can affect B-cell differentiation into autoantibody-producing plasmablasts in autoimmunity. Alterations in the activation of immunocytes caused by omega-3 fatty acids (a family of polyunsaturated fatty acids, PUFAs) have been described for many years (136). Supplementation with omega-3 FAs could reduce autoantibody production and immunocomplex deposition, and hinder interferon and chemokine gene expression in lupus (137, 138). Consistently, dietary short-chain FAs can regulate B cell differentiation, which critically underpins effective T-dependent and T-independent antibody responses in lupus-prone mice (139). Overall, these findings support the hypothesis that dietary supplementation with specific fatty acids can attenuate lupus disease activity by affecting B cells.

### Lipid Metabolism in Innate Immune Cells in SLE

Macrophages are also important in the regulation of SLE by engulfing various lipids through phagocytosis. Nitro-fatty acids, reactive lipid species produced by metabolic and inflammatory reactions, can modulate immune cell functions, thus exerting an influence on various pathologies, particularly inflammation (140). Moreover, nitro-fatty acids can modulate the process of macrophage differentiation and adhesion to endothelial cells and their chemotaxis (141). Notably, the ox-LDL uptake through macrophage scavenger receptors is a key initial process in the formation of foam cells, which is a critical precursor to atherosclerosis (142). Interferon-α priming can enhance the uptake of ox-LDL in macrophages, which promotes the formation of foam cells *in vitro*, and monocytes from patients with SLE display higher lipid uptake (143). In a lupus-prone mouse model, FcgRIIB knockout (KO) mice, dysregulation of lipid metabolism in macrophages was found to be responsible for lipopolysaccharide (LPS) tolerance, such as higher levels of phosphatidylethanolamine (PE), a class of phospholipids in biological membranes found in their macrophages (144). Exploring approaches to regulate lipid metabolism is a potential strategy to harness LPS tolerance in lupus. Transcriptional regulation through PPARγ is essential for changes in lipid metabolism during monocyte differentiation into macrophages. Blocking PPARγ-dependent signaling during monocyte differentiation could result in a macrophage phenotype characterized by attenuated reduced inflammatory responses to oxidized lipoproteins and saturated FAs, which could benefit patients with cardiometabolic disorders (145). Agents targeting PPARγ can promote the differentiation of monocytes towards an M2 phenotype and ameliorate the condition of SLE patients (19). The enzyme 12/15-PG (12/15-LO) oxygenates free and phospholipid-bound PUFAs. The expression of 12/15-LO is induced by the stimulation with some interleukins and is restricted to certain macrophages. It has been revealed that defective 12/15-LO activity led to an abnormal monocyte phagocytosis, subsequent antigen presentation of aberrant antigens derived from apoptosis, and a lupus-like autoimmune disease (146). Defects in eliminating dying cells contribute to the accumulation and secondary necrosis of apoptotic cells, which release signals such as S1P to induce phagocytic removal. S1P, a mediator produced from membrane sphingolipids, could regulate trafficking of adaptive immunocytes (147). Dying cell-derived S1P activates erythropoietin (EPO) signaling in macrophages, then enhances clearance of dead cells through PPARγ signaling. Consistently, macrophage-specific Epor(-/-) mice could develop lupus-prone models, the condition of which was improved by interference with S1P-EPO signaling (148). Liver X receptors (LXRs) play pivotal roles in the transcriptional control of cholesterol and lipid metabolism; its isotype, LXRα is extensively expressed in macrophages. Several animal models of atherosclerosis have demonstrated that stimulating LXRs could reduce disease development, which could be attributed in part to the capability of LXRs to facilitate macrophage cholesterol efflux (40). LXRα gene promoter polymorphisms are reported to be related to Korean SLE patients (41). A study assessed cytokine expression related to LXRα polymorphisms in monocyte-derived macrophages obtained from SLE patients and found that higher levels of pro-inflammatory cytokines are associated with the expression of Toll-like receptors (149). Overall, LXRs play complicated roles in macrophages and can connect the inflammatory response and lipid metabolism in SLE. Furthermore, it has been reported that in lupus-prone mice, injuries of end-organs could be rescued by an agonist of LXR (150). Hence, future investigations focusing on the role of LXRs in pharmacotherapy of patients with SLE are warranted.

Dendritic cells (DCs) are known for their ability to link innate and adaptive immune processes, and their uncontrolled activation drives autoimmunity (151). DCs can sense cytokines, danger signals, and lipid species such as saturated fatty acids and ox-LDL. Altered lipid concentrations or stimulation by lipids can modulate the functions of DCs (152). The direct administration of LDLs and ox-LDL to DCs could promote the production of IL-6, which in turn would enhance susceptibility to autoimmune diseases by regulating pathogenic autoimmune Th17 polarization and inflammatory functions (153). Lipid profiles that reflect atherosclerosis have been proven to lead to local activation of murine DCs in the skin, promote dermal inflammation, and induce lymph node hypertrophy (154). Cholesterol efflux mediated by HDL is dependent on the ATP-binding cassette transporters A1 and G1 (ABCA1/G1). Notably, mice deficient in ABCA1/G1 in DCs develops autoimmune nephritis; DC specific deficiency of ABCA1/G1 enhances the activation of T cells, as well as the polarization of Th1 and Th17 cells; cholesterol-enriched DCs shows augmented cytokine secretion and activation of inflammasomes (42). Increased expression of type 1 interferon (IFN)-regulated genes is considered a hallmark of SLE, and human plasmacytoid DCs are important in SLE pathogenesis through enhanced IFNα (155). Mitochondrial mitochondrial import of pyruvate and FA synthesis are important to the increased FAO in IFN-activated plasmacytoid DCs, and these changes in lipid metabolism are PPARα-dependent and critical to cell and cytokine functions (20). Given the importance of IFNs in SLE, there is a clinical need to explore approaches such as metabolic targeting as a potential adjunct to conventional treatment strategies.

Neutrophils can interact with various immunocytes and are implicated in the pathogenesis of inflammatory and autoimmune diseases (156). A distinct subset of lupus proinflammatory neutrophils (LDGs) has been found to increase the risk of CVD in SLE (157). The noncalcified plaque burden enhances the prevalence of high-risk plaques in some inflammatory conditions. Accordingly, the noncalcified plaque burden was found to be elevated in SLE, and was associated with LDGs and cholesterol efflux capacity. Neutrophils can undermine the function of HDL, thereby promoting atherogenesis (158). Substantial interest has been given to study the involvement of heightened levels of oxidative stress in SLE, which contributes to dysregulated immune system. In one study, a cafeteria diet-fed lupus-prone mice showed increased cholesterol levels, and neutrophils exhibited enhanced ROS production capacity, which may contribute to accelerated SLE onset (159). Moreover, SLE patients showed increased ROS production and enhanced catalase activity in neutrophils. Neutrophils are characterized by lower levels of Malondialdehyde (MDA), a lipid peroxidation biomarker, and high levels of protein oxidation (160). The oxidation of cholesterol and phospholipids containing PUFA chains can contribute to the lipid peroxidation, in which lipid hydroperoxides are critical intermediates. Mechanistically, inhibition of glutathione peroxidase 4 (GPX4) results in lipid peroxidation and induction of ferroptosis, a regulated cell death featured by the aggregation of lipid ROS dependent on the iron (43).

Recently, a study found that mice with neutrophil-specific GPX4 haploinsufficiency recapitulated the critical clinical manifestation of SLE patients, and the disease activity in mouse models could be obviously ameliorated by a specific ferroptosis inhibitor (44). Current evidence suggests that neutrophils in patients show the ability to form neutrophil extracellular traps (NETs), which could stimulate the production of IFNs and multiple cytokines. NETs possess oxidant-generating enzymes such as myeloperoxidase. Experiments confirmed that active oxidant-generating enzymes within NETs, such as MPO, could modify HDL and render lipoprotein proatherogenic (161). Taken together, these findings suggest an emerging role for NET-derived lipid oxidation in SLE-relevant atherosclerosis, which could be targeted in future therapies.

## Targeting Lipid Metabolism Associated with Immunity for SLE Treatment

Current drugs used for SLE treatment include various immunosuppressive agents that can cause adverse reactions. As lipid-associated metabolic dysregulation has been implicated in the pathogenesis and progression of SLE and/or complications, including lupus nephritis and cardiovascular diseases, targeting lipid metabolism is a potential adjunct treatment in these situations. Notably, steroids, which are widely used in patients with SLE, can alter metabolite profiles and increase cardiovascular risk. Glucocorticoids have been reported to exhibit metabolic effects by increasing the production of leptin, which may activate mTOR (162). Immunosuppressive drug, mycophenolate mofetil (MMF), could prevent SLE-associated CVD by inhibiting CD4+ T cell activation and infiltration into atherosclerotic lesions, which was accompanied by a decline in IgG1 serum titers to ox-LDL (163). Therefore, treatment with MMF alone or in combination with statins may benefit patients with SLE and atherosclerosis. The B cell-activating factor (BAFF) receptor pathway is of importance for the promotion of B-cell differentiation and survival of plasma cells. Overexpression of BAFF in the model of transgenic mouse increases lupus-like autoantibodies in B cells (164). In 2011, the US Food and Drug Administration approved an antibody against soluble BAFF as a treatment for patients with SLE. A study demonstrated that inhibiting the BAFF receptor ablated B-2 cells and reduced the frequency of mouse atherosclerosis (165). BAFF blockade could be expected to lower the cardiovascular risk in patients with SLE. IFN, low-density neutrophils (LDNs), and NETs are now considered as potential key players in SLE-associated vascular damage. The Janus kinase (JAK) inhibitor tofacitinib has been demonstrated to improve immunity and vascular disorders in lupus-prone models by exerting effects on immune cells and cytokines (166). Recently, a phase 1 double-blind randomized trial of tofacitinib demonstrated to improve HDL-c levels, particle numbers, and cholesterol efflux capacity. The use of tofacitinib significantly reduced the type I IFN gene signature, levels of LDNs and circulating NETs, which consequently ameliorated SLE and its associated vascular dysfunction (167). In addition, baricitinib, another JAK inhibitor that controls intracellular signaling induced by cytokines such as IL-6 and IFN, was proven more effective in ameliorating clinical manifestations of SLE patients than placebo in a phase IIb clinical trial (168). Considering the role of PPARγ in the pathogenesis of SLE, agents targeting PPARγ may serve as adjuvants for the treatment of SLE. Pioglitazone, a PPARγ agonist, regulates CD4+T cell function and modulates the differentiation of monocytes and monocyte-derived macrophages, as previously reported in lupus (19, 21). Moreover, another PPARγ agonist, rosiglitazone, downregulates the production of autoantibodies and prevents atherosclerosis and lupus nephritis (22). The disruption of CD40 signaling influences atherogenesis in hyperlipidemic mice (169). PPARγ modulates inflammatory signals induced by the activation of CD40, which complicates the mechanisms of PPARγ in SLE (23). Combined with glucocorticoids, rosiglitazone induced stable tolerogenic dendritic cells in monocytes derived from patients with SLE (170). Taken together, PPAR-γ agonists could be further explored as potential treatments for SLE. Statins exert beneficial vascular effects in SLE through mechanisms including inhibition of cholesterol biosynthesis and immunomodulatory functions. Monocytes collected from patients with SLE were treated *in vitro* with fluvastatin, and cells showed altered mitochondrial membrane potential and higher oxidative stress, and *in vivo* fluvastatin treatment improved the condition of patients with reduced lipid levels and vascular inflammation (171). Antiphospholipid antibodies (aPL) (occur in 30–40% of patients with SLE) are associated with increased risk of atherosclerosis as well. A prospective open-label pilot study demonstrated that some biomarkers, including TNF-α and soluble CD40L, upregulated in aPL-positive patients can be reversibly reduced by fluvastatin (172). Lipidomics analysis of SLE showed that alterations in lipids in peripheral blood mononuclear cells could be improved after treatment with an antioxidant *in vitro*, and the levels of pro-inflammatory cytokines as well as IgG autoantibodies were significantly reduced after treatment *in vivo* (83).

Omega-3 fatty acids, which was proven to reduce SLE activity, should also be considered in addition to the conventional regimens used for SLE (173). It is emerging that the excessive release of STING-dependent type I IFNs is implicated in certain interferonopathies, including SLE. Nitro-fatty acids can affect the release of type I IFN by modifying STING-associated signaling. Besides, endogenously formed nitro-fatty acids can serve as inhibitors of the STING-IFN axis. Further research on the novel roles of these lipids in the treatment of SLE is needed (174). Overall, understanding the mechanisms of lipid metabolism in SLE, particularly in pathogenic immunocytes, offers an opportunity to develop novel adjunct therapies to ameliorate progression of lupus and its related complications.

## Conclusion and Future Perspectives

In this review, we illustrated a complicated lipid metabolic network in SLE. This highlights that lipid synthesis, oxidation, degradation, metabolites, and related signaling processes are implicated in immunocyte activation, differentiation, and immune responses. Further investigation is required to develop new approaches targeting lipid metabolism to treat patients with SLE. Lipogenesis is a major target for treatment because cells rely on lipids that provide energy and related signaling for their basic activities. Moreover, immunocytes rely on lipids to exert their specific functions in response to stimuli; thus, balancing the effects of lipid metabolism on immunocytes and the internal environment is necessary to explore adjunct targets. Nevertheless, certain questions and challenges, such as metabolism being tissue-or organ-specific, and many aspects of lipid metabolic changes may even be unique to distinct immunocyte types, not to mention their sophisticated complexity at the biochemical level, remains to be addressed Although there are promises for clinical translation, the results of the emerging therapies are variable. Moreover, the mechanisms of lipid metabolism acting directly or indirectly on SLE are complex, and multiple factors have been implicated. The involvement of lipids in the immune system, especially among different cell populations, emphasizes the need to study the effects of interfering with lipid metabolism in suitable *in vivo* models, particularly in combination with conventional immunosuppression therapies. Rational strategies for SLE therapies should be further explored owing to the availability of agents targeting various parts of lipid metabolism. Lipidomics, an approach to provide global profiles of lipids, has improved significantly with the advancement of next-generation mass spectrometry instruments, and bioinformatics; all of which promoted lipid biology to the forefront of metabolism studies (175). Insights into the regulation of lipid metabolism may provide novel therapeutic strategies for SLE treatment.

## Author Contributions

YL and YS contributed to support the conception of the review. WS and PL contributed to writing the manuscript and making figures. JC, JM, and XZ read, discussed, and revised the manuscript. All authors listed have approved the submitted version and publication.

## Funding

This work was supported by National Natural Science Foundation of China (81788101, 81630044), Chinese Academy of Medical Science Innovation Fund for Medical Sciences (CIFMS) (2021-1-I2M-017, 2021-1-I2M-047, 2021-1-I2M-040, 2021-1-I2M-016, and 2021-1-I2M-026), and Capital’s Funds for Health Improvement and Research (2020-2-4019).

## Conflict of Interest

The authors declare that the research was conducted in the absence of any commercial or financial relationships that could be construed as a potential conflict of interest.

## Publisher’s Note

All claims expressed in this article are solely those of the authors and do not necessarily represent those of their affiliated organizations, or those of the publisher, the editors and the reviewers. Any product that may be evaluated in this article, or claim that may be made by its manufacturer, is not guaranteed or endorsed by the publisher.

## Glossary

**Table:**

SLE	systemic lupus erythematosus
DCs	dendritic cells
FA	fatty acid
FAs	fatty acids
FASN	fatty acid synthase
ACC	acetyl-CoA carboxylase
HMGCR	3-hydroxy-3-methylglutaryl CoA reductase
GSL	glycosphingolipid
PI3K	phosphoinositide 3-kinase
LXR	liver X receptor
SREBP	sterol regulatory element-binding protein
PPAR	peroxisome proliferator-activated receptor
ROS	reactive oxygen species
FAO	fatty acid oxidation
TCA	tricarboxylic acid
LDs	lipid droplets
LDL	low-density lipoprotein
TG	triglyceride
TC	total cholesterol
HDL	high-density lipoprotein
ApoA-1	apolipoprotein A-1
ApoB	apolipoprotein B
LN	lupus nephritis
NF-κB	the nuclear factor κB
PON-1	paraoxonase-1
SLEDAI	Systemic Lupus Erythematosus Disease Activity Index
CVD	cardiovascular disease
ox-LDL	oxidized LDL
TCR	T-cell antigen receptor
Fli1	Friend leukaemia integration 1
Th	T helper
T	Treg, T regulatory
RICD	restimulation-Induced Cell Death
S1P	sphingosine 1-phosphate
IFN-γ	interferon-γ
MDA	malonaldehyde
AMPK	AMP-activated protein kinase
iNKT	invariant natural killer T
BTLA	B and T lymphocyte attenuator
BCR	B cell receptor
AtMs	atypical memory B cells
PUFA	polyunsaturated fatty acids
EPO	erythropoietin
ABCA1/G1	ATP Binding Cassette Transporters A1 and G1
LDGs	low density granulocytes
GPX4	glutathione peroxidase 4
NETs	neutrophil extracellular traps
MMF	Mycophenolate mofetil
BAFF	B cell-activating factor
LDNs	low-density neutrophils
JAK	Janus Kinase
aPL	antiphospholipid antibodies
